# Infection-related Glomerulonephritis and C3 Glomerulonephritis - Similar Yet Dissimilar: A Case Report and Brief Review of Current Literature

**DOI:** 10.7759/cureus.7127

**Published:** 2020-02-28

**Authors:** Gajapathiraju Chamarthi, William L Clapp, Harini Bejjanki, Jena Auerbach, Abhilash Koratala

**Affiliations:** 1 Nephrology/Renal Transplantation, University of Florida Health, Gainesville, USA; 2 Pathology, University of Florida Health, Gainesville, USA; 3 Nephrology, University of Florida Health, Gainesville, USA; 4 Nephrology, Medical College of Wisconsin, Milwaukee, USA

**Keywords:** infection-related glomerulonephritis, post-streptococcal glomerulonephritis, c3 glomerulonephritis, alternate pathway complement, membranoproliferative glomerulonephritis (mpgn)

## Abstract

Infection-related glomerulonephritis (IRGN) is an immune complex-mediated glomerulonephritis (GN), often preceded by infection with subsequent recovery of renal function after the resolution of the infection. C3 deposition in the absence of immune complex deposits can be seen in patients with IRGN, but with the emergence of C3 glomerulonephritis (C3GN), the distinction is difficult as the clinical and pathological presentation may be similar. However, their treatment and clinical course vary significantly.

A 64-year-old man with a history of hypertension and bioprosthetic aortic valve presented to the Emergency Department with left upper quadrant (LUQ) pain and a purpuric rash on bilateral lower extremities. The patient became septic, and further workup during the hospitalization revealed endocarditis secondary to Streptococcus viridans. On admission, the patient had acute kidney injury (AKI) with a serum creatinine of 3.79 mg/dl, which peaked at 5.72 mg/dl during the hospitalization. Renal biopsy demonstrated segmental necrotizing glomerulonephritis on light microscopy, predominant C3 deposition on immunofluorescence (IF) staining, and mesangial and paramesangial deposits on electron microscopy. This histologic picture can be seen both in IRGN and C3GN. The patient was treated with intravenous ceftriaxone for six weeks for endocarditis and the kidney injury was managed with supportive care. The patient’s renal function improved and complement levels normalized, supporting the diagnosis of IRGN retrospectively. IRGN can mimic C3GN, and evaluation for alternate pathways of the complement system may be warranted in patients with atypical presentation of IRGN.

## Introduction

Infection-related glomerulonephritis (IRGN) is an immune complex-mediated injury to the host glomeruli, which is usually triggered by an extrarenal infection. Post-streptococcal infectious glomerulonephritis (PSGN) has been described as a classic example of IRGN, seen in children as a sequel to a recent upper respiratory tract infection or impetigo [1]. The epidemiology, demographics, and the incidence of IRGN have changed over the past decades. PSGN is still the most common cause of glomerulonephritis (GN) in children with approximately 472,000 reported cases per year, but the incidence has decreased considerably in developed countries due to improved socioeconomic conditions and early access to health care and antibiotics [2]. The clinical features and biopsy findings of IRGN can vary significantly from the classically described PSGN. With emerging entities such as C3 glomerulonephritis (C3GN), the histologic variants of IRGN can pose a diagnostic and therapeutic challenge to the treating physician. The clinical and pathological features overlap between IRGN and C3GN. The renal biopsy findings often do not guide us to a specific diagnosis. Diagnosing C3GN requires testing for complement pathways, which are usually cost-prohibitive in some countries, and turnaround time for some of these tests can take weeks, leaving the treating physician with a diagnostic conundrum [3]. Treating the infection usually results in the resolution of IRGN, whereas no robust guidelines exist in the treatment of C3GN. Therapies targeting complement pathways, such as eculizumab, and immunosuppressive agents, such as mycophenolate and prednisone, have shown to induce remission in a few case series. However, results have not been consistent across the board [4]. The prognosis is poor in patients with C3GN and 50% of the adult patients’ progress to end-stage renal disease (ESRD). There is also a high predilection for recurrence and graft failure in post-transplant patients [5]. We present a case of endocarditis-associated glomerulonephritis with features suggestive of C3GN on renal biopsy.

## Case presentation

**Figure 1:**
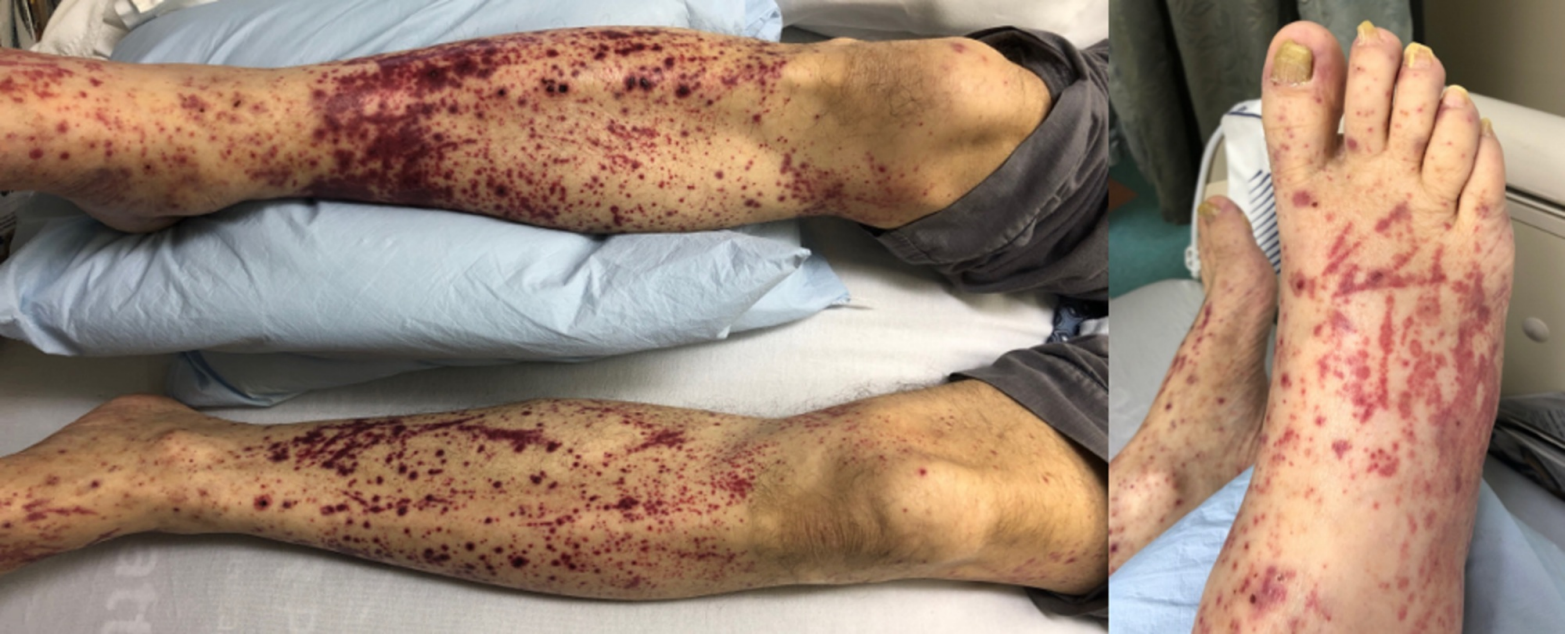
Photograph demonstrating the purpuric rash on the patient’s bilateral lower extremities at presentation.

A 64-year-old male construction worker presented to the emergency department (ED) with left upper quadrant (LUQ) abdominal pain and a rash over the bilateral lower extremities for three days. The patient complained of LUQ pain radiating to the left flank and a dark red-colored rash involving both lower extremities that he noticed three days before the presentation. Associated symptoms included night sweats and worsening nausea. Besides, he reported a 40-pound weight loss over the past few months and attributed it to intermittent nausea and a decrease in appetite. He denied having chest pain, shortness of breath, fevers, chills, or sick contacts. He had no hematuria and denied using any antibiotics, non-steroidal anti-inflammatory drugs (NSAID), or over-the-counter or herbal medication in the recent past. He denied any joint pain or swelling, sinus disease, foot drop, epistaxis, Raynaud's phenomenon, digital ulcers, or hemoptysis. His past medical history was significant for ascending aortic aneurysm repair, aortic valve replacement (secondary to the bicuspid aortic valve and severe aortic stenosis), and hypertension. His only medication before admission was metoprolol, 50 mg twice daily. On initial examination in the ED, the patient was afebrile with a documented temperature of 36.7°C. He was hemodynamically stable with a blood pressure of 113/66 mmHg and pulse rate of 72 beats/min. He had a systolic murmur in the aortic area. Abdomen examination was significant for mild tenderness in the left upper quadrant with no guarding or rigidity. He was noted to have a non-blanching, erythematous, petechial rash in the lower extremities (Figure 1).

**Figure 2:**
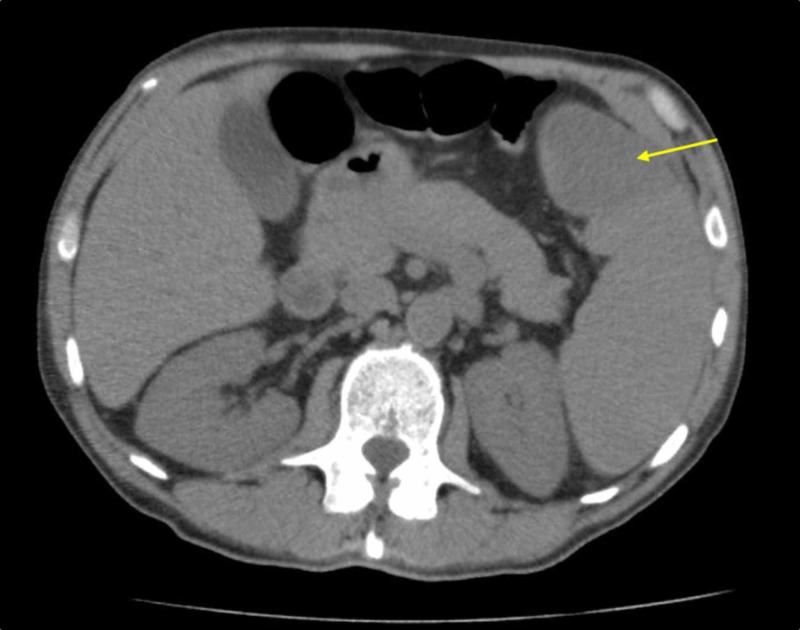
Computed tomography (CT) scan of abdomen/pelvis showing splenomegaly with a hypodense region (yellow arrow) suggestive of a splenic infarct

Initial laboratory testing was significant for a blood urine nitrogen (BUN) of 58 (6 - 21 mg/dL), serum creatinine 3.79 (0.51 - 1.18 mg/dL), and bicarbonate 19 (22 - 30 mmol/L). Aspartate aminotransferase (AST) was minimally elevated at 45 (0 - 37 IU/L), while alanine aminotransferase (ALT), lipase, and bilirubin levels were within the normal limits. Other notable laboratory values were a hemoglobin of 8.1 (13 - 16.5 g/dL), mean corpuscular volume (MCV) of 84.3 (78 - 100 fl), and platelet count of 171,000 (150 - 450 thousand/cu mm). Urinalysis showed 172 red blood cells/high power field (RBC/hpf) and 2+ protein on the dipstick. In the ED, the patient underwent computed tomography (CT) imaging of the chest, abdomen, and pelvis without contrast, which showed splenomegaly with hypodense splenic region measuring up to 5.7 cm, suggestive of a splenic infarct (Figure 2).

**Figure 3:**
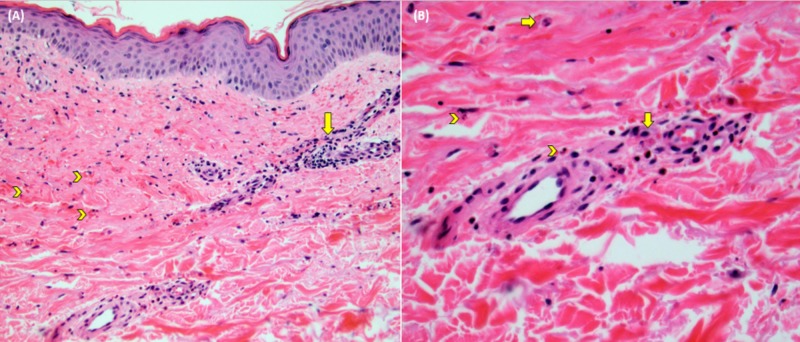
Skin biopsy demonstrating (A) perivascular inflammation (arrow) and extravasated red blood cells (chevrons) in the low-power view (20x) and (B) perivascular and interstitial neutrophils (arrows) and eosinophils (chevrons) in the high-power view.

Renal ultrasound demonstrated the right kidney measuring 12.6 x 5.3 x 5.8 cm, left kidney measuring 15.3 x 5.5 x 5.9 cm with normal urinary bladder, and absence of hydronephrosis. The patient clinically deteriorated in the next few hours and was found to have sepsis. He was started on broad-spectrum antibiotics (vancomycin and cefepime), which was later changed to ceftriaxone as per the sensitivities. Blood cultures were positive for Streptococcus viridans, and transesophageal echocardiogram (TEE) was suggestive of infective endocarditis of the aortic and mitral valves. Serology workup, including anti-nuclear antibody (ANA), antineutrophil cytoplasmic antibodies (ANCA), anti-glomerular basement membrane (anti-GBM) antibodies, hepatitis B, and hepatitis C, were negative; rheumatoid factor and cryoglobulin tests were positive. Serum complements levels were low with C3 at 77 (87 - 200 mg/dl) and C4 at 11 (13 - 50 mg/dL). Kappa/lambda light chain ratio was normal, and serum protein electrophoresis showed no M spike or abnormal bands. Skin biopsy showed pathology consistent with leukocytoclastic vasculitis (Figure 3).

The patient’s baseline serum creatinine was 1.06 mg/dL a month before his presentation. The creatinine on admission was 3.79 mg/dL, which peaked at 5.77 mg/dL during hospitalization. A 24-hour total protein excretion was ~1.1 g (ref: < 150 mg). Given the findings of infective endocarditis, purpuric rash, and positive serology for cryoglobulins, the initial differential diagnosis was broad, including acute tubular necrosis (ATN), IRGN, cryoglobulinemic vasculitis, and ANCA vasculitis.

**Figure 4:**
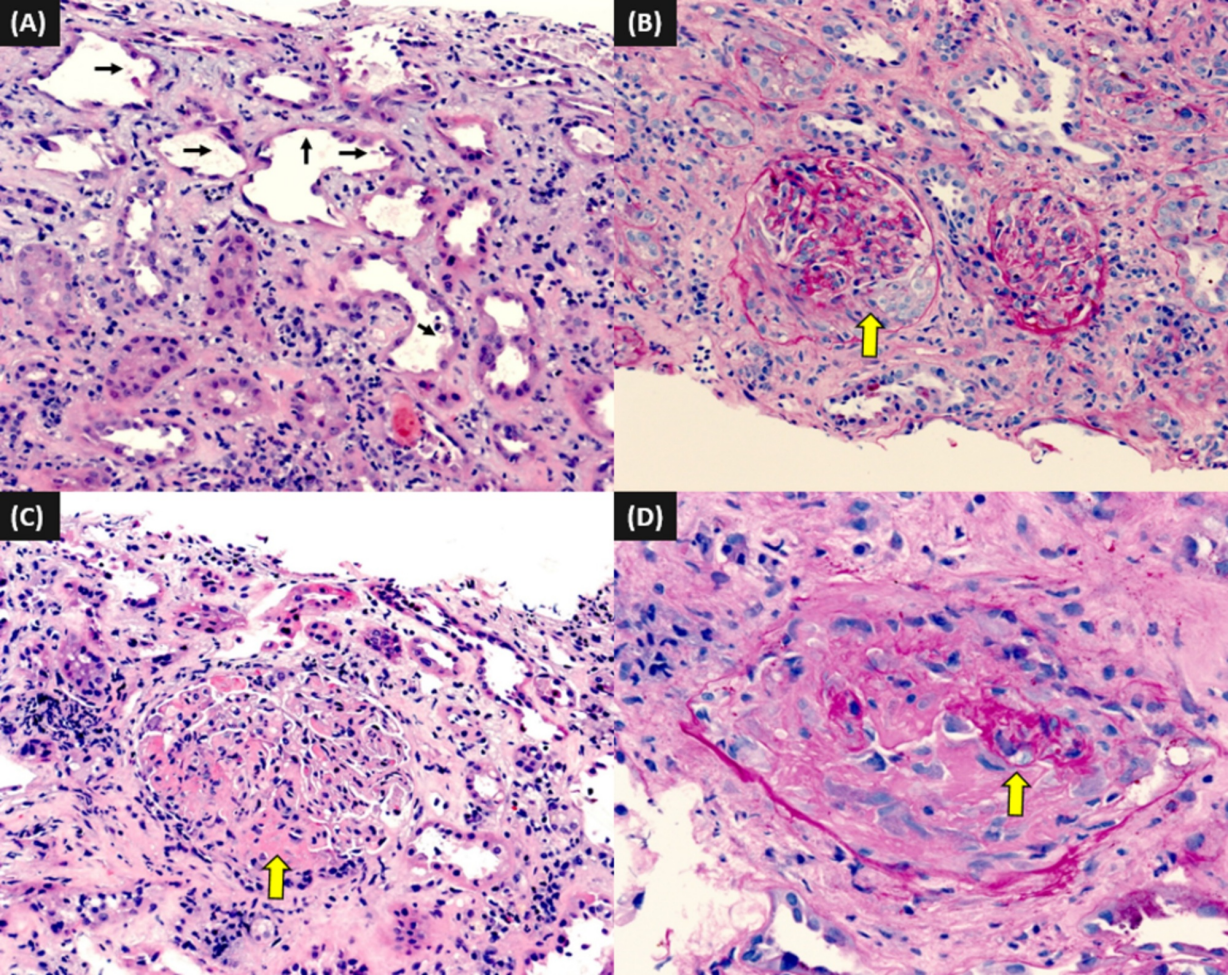
Light microscopy images from the renal biopsy (A) acute tubular injury (arrows) (hematoxylin & eosin (H&E) 400x); (B) a glomerulus with segmental cellular crescent (arrow) (periodic acid-Schiff (PAS), 200x); (C) a glomerulus with segmental necrosis (arrow) (H&E, 200x), (d) a glomerulus obliterated by fibrinoid necrosis (arrow) (PAS, 400x)

**Figure 5:**
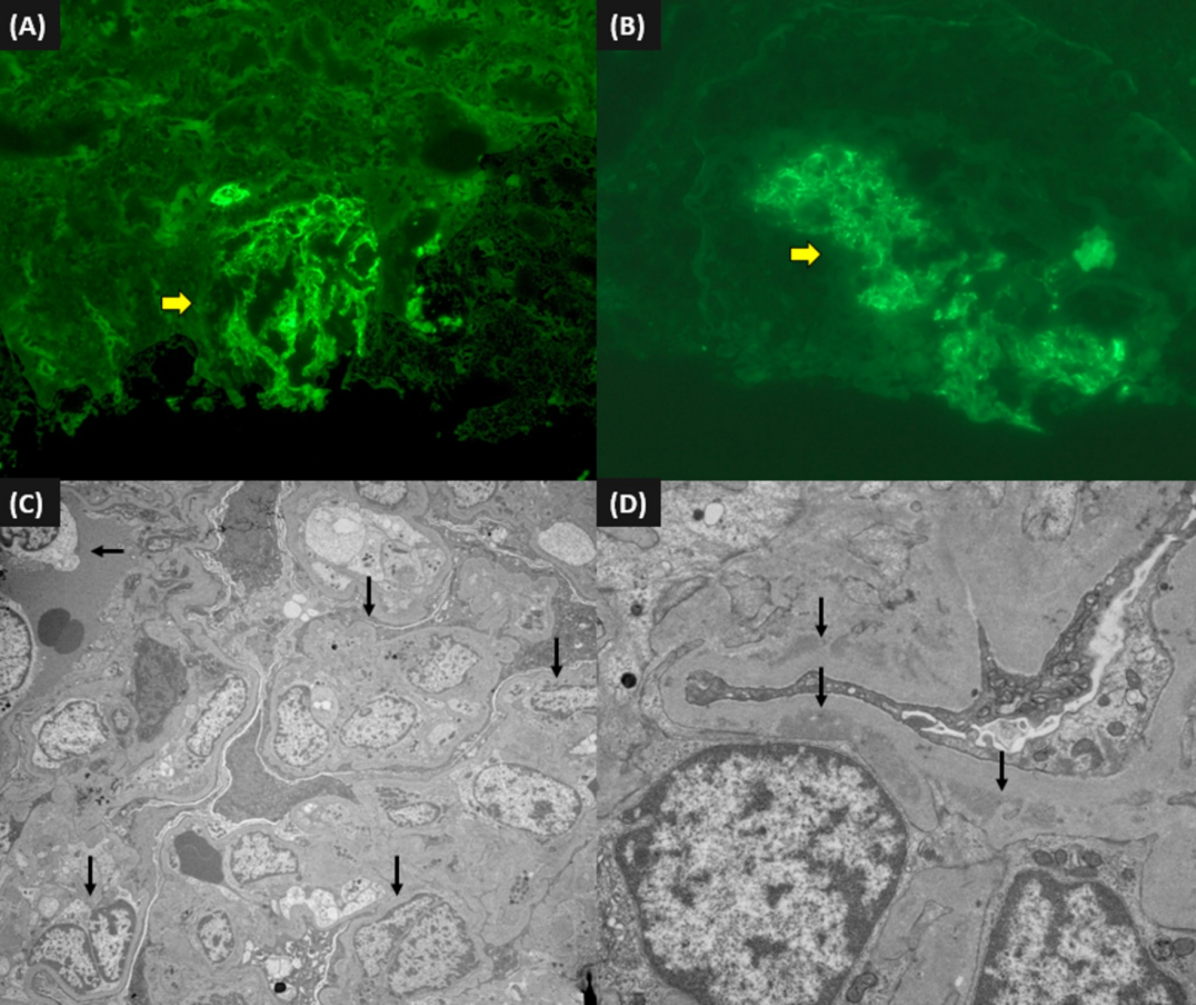
Immunofluorescence microscopy from the renal biopsy (A) glomerular staining for C3 (200x); (B) a glomerulus compressed by crescent showing C3 deposition (arrow) (400x); (C) electron microscopy demonstrating glomerular hypercellularity (arrows) (3,000x); (D) mesangial and paramesangial electron dense deposits (15,000x)

Renal biopsy was pursued, which showed findings of crescentic glomerulonephritis and severe acute tubular necrosis (ATN). Light microscopy showed segmental cellular crescents in the glomeruli and ATN characterized by tubular dilatation, epithelial attenuation, and cell sloughing, necrotic debris, and red cells in the tubular lumen. Immunofluorescence (IF) demonstrated only C3 deposits (2+) and was negative for IgG, IgM, IgA, C1q, albumin, kappa, and light chain. Electron microscopy (EM) showed increased mesangial cellularity and glomerular basement membrane (GBM) thickness with deposits predominantly seen in the mesangium and paramesangial area (Figures 4-5].

The differentials included post-infectious GN or C3GN. Given the degree of ATN on the biopsy and the clinical findings suggestive of possible IRGN, the patient was monitored for his AKI with conservative measures. The patient showed signs of renal recovery with improvement in urine output, and the serum creatinine trended down to 4.63 mg/dL on the day of discharge. 

The patient completed a six-week course of intravenous ceftriaxone for the endocarditis, and at follow-up, the AKI had improved with a serum creatinine of 1.90 mg/dL and urine protein-creatinine ratio of 350 mg/g (ref: < 150 mg/g). Repeat urinalysis showed zero RBC/hpf. Of note, C3 and C4 normalized with C3 at 110 mg/dL and C4 at 33 mg/dL.

## Discussion

The spectrum of IRGN has changed over the last few decades, especially in developed countries, with IRGN being predominantly seen in the elderly with comorbidities, such as diabetes mellitus, intravenous drug use, alcoholism, malignancy, or immunosuppressive states [6]. The term IRGN has been proposed as patients usually have ongoing contaminant infection, and the findings on pathology seem to differ from the classic PSGN [7]. Upper respiratory tract infections, skin infections, pneumonia, and endocarditis are common sources for IRGN with Staphylococcus and Streptococcus being the major pathogens isolated. While several cases of IRGN associated with gram-negative organisms or fungal pathogens have been reported, one-third of cases with classical IRGN had no isolated organism [1, 6-7].

On renal biopsy, the classic cases of PSGN usually manifest as proliferative GN on light microscopy, immunoglobulin, and C3 deposition on immunofluorescence (IF), appearing as a starry sky or garland appearance and subepithelial humps on electron microscopy (EM) [8]. The term "infection-related glomerulonephritis (IRGN)" is preferred in patients who have an ongoing infection, and the typical pattern on light microscopy is endocapillary proliferation. Patients with infective endocarditis and associated GN can have varied histology and can present from classical diffuse endocapillary proliferation, as seen in PSGN, to a crescentic pattern [9]. In a recent clinicopathological series in patients with infective endocarditis (IE) by Boils et al., crescentic GN was the most common pattern, followed by diffuse proliferative GN. Acute tubular necrosis (ATN) was present in 86% of cases, C3 was present in 94% of cases, and C3 without immunoglobulin deposition was present in 37% of the cases; only 14% showed classic subepithelial hump-like deposits on electron microscopy [10].

Membranoproliferative glomerulonephritis (MPGN) was previously classified as MPGN I characterized by predominant subendothelial deposits, MPGN II characterized by intramembranous deposits, and MPGN III characterized by subepithelial and subendothelial deposits. With a better understanding of the pathophysiology of glomerular diseases, MPGN was recently classified into immune complex or predominantly complement driven disease states [11]. Mass spectrometry and proteomic analysis of the deposits have helped in organizing the MPGN and introducing the term C3 glomerulopathies. C3 glomerulopathies have predominant C3 deposition of higher than twofold magnitude with absence or trace amount of immunoglobulin deposition. They are subclassified into C3 glomerulonephritis (C3GN) and dense deposit disease (DDD) based on the EM findings. Uncontrolled activation of the complement alternative pathway (CAP) driven by genetic or acquired defects plays a predominant role in the pathogenesis of these diseases. It often requires testing for complement activity for the diagnosis of the disease. The tests have a turnaround time of weeks and are not widely available [12]. Further, the tests may not help the clinician or pathologist in making a definitive diagnosis in the acute setting.

The light microscopy findings can have varied histology in the C3GN with membranoproliferative being the most common pattern but can show mesangial or diffuse endocapillary proliferation patterns [13]. C3 staining on the immunofluorescence greater than two-fold in intensity in comparison to the other immune proteins is essential to make the diagnosis of C3 glomerulopathies [5]. The EM findings help to differentiate between DDD and C3 glomerulonephritis. Highly osmiophilic ‘sausage-shaped’ deposits in the glomerular basement (GBM) are characteristic of DDD, whereas, in C3GN, the deposits are often lighter in appearance compared to DDD and predominantly seen in the mesangium and subendothelial regions [9, 14].

The clinical and pathological features can overlap between IRGN and C3GN, with infection as the known triggering factor for IRGN, which can also be an inciting factor for the C3GN. In a retrospective analysis of biopsy series, antistreptolysin-O (ASO) was elevated in 57% of C3GN cases, suggesting streptococcal infection as the preceding infection [15]. Both IRGN and C3GN can often present with hematuria and proteinuria. Biopsy findings of endocapillary proliferation with subepithelial humps, which are classically seen in IRGN, can be present in C3GN as well [13, 15]. Case reports of C3 glomerulopathy presenting as crescentic nephropathy have also been reported in the literature [16]. C3 levels may be normal in some patients with C3GN. As mentioned earlier, in a series of infective endocarditis patients with GN, C3 only deposition was seen in 37%. In the resolving phase of PSGN, C3 is often the predominant deposition without immunoglobulin. A consensus group that came up with the criteria for defining C3GN does note that differentiating between IRGN and C3GN can be difficult, and the definitive diagnosis often requires following the patients clinically and serologically. Many authors suggest investigating for complement abnormalities in a patient who does not follow the typical course of IRGN [17]. Normalization of the complement levels within eight to 12 weeks after infection and the absence of recurrence of hematuria and proteinuria often suggest IRGN, as was the case with our patient. Patients with clinical features suggestive of atypical pauci-immune glomerulonephritis (PIGN), such as persistent low complements levels, recurrence of symptoms of GN, such as proteinuria and hematuria, or a family history of GN, should prompt the clinician to evaluate for C3 glomerulopathies. The workup often involves testing for the C3 nephritic factor, CH50 (classic hemolytic pathway hemolytic essay), AH50 (alternate pathway hemolytic essay), measurement of Factor H and Factor I, serum paraprotein detection, and anti-factor H autoantibodies. Genetic testing for mutations in complement regulators (complement factor H (CFH), complement factor I (CFI), complement factor B (CFB), membrane cofactor protein (MCP), and C3) may have to be evaluated based on a case by case basis [3]. Our patient had a light microscopy finding of crescentic glomerulonephritis, but given the findings on IF and EM, C3GN was also high in our differential. The overall constellation of clinical features, improvement in AKI, normalization of C3 and C4, and resolution of hematuria was consistent with infective endocarditis-associated GN in our patient. Testing for alternative complement pathways was not undertaken in our patient due to the improvement of renal functions, the normalization of complement levels, and the resolution of proteinuria and hematuria.

Treating the infection usually results in the resolution of IRGN, whereas no robust guidelines exist in the treatment of C3GN. Therapies targeting complement pathways, such as eculizumab, and immunosuppressive agents, such as mycophenolate and prednisone, have shown to induce remission in case series. However, results have not been consistent across the board [4, 18]. Mycophenolate mofetil has been recommended for the treatment of moderate disease (described as urine protein over a 500 mg/24 hour or moderate inflammation) as per the recent Kidney Disease: Improving Global Outcomes (KDIGO) controversies conference [19]. No specific recommendations were made for severe disease (defined as urine protein over a 2,000 mg/24 hour despite immunosuppression or severe inflammation represented by endocapillary proliferation associated with or without crescents) due to lack of insufficient data. The prognosis of C3GN is poor, with approximately 50% of the adult and 70% of the pediatric populations progressing to ESRD over 10 years [5]. Few case series have looked at the outcomes of C3GN patients after renal transplantation. The recurrence rate is high, ranging from 55% to 86%, despite patients being on immunosuppressive medications. Graft failure was noted to vary from 30% to 50% in C3GN to 83% in DDD [20].

## Conclusions

We report our case to highlight the dilemma faced by nephrologists and nephropathologists in the diagnosis and management of IRGN and C3 glomerulopathy. We want to reiterate the importance of serial follow-up of patients and the need for further workup in patients with an atypical course of IRGN, such as a progressive decline in renal function, the persistence of hypocomplementemia, proteinuria, or hematuria. Even in classic cases of PIGN, follow-up is needed to ensure that there is a resolution of renal failure and an absence of recurrence of symptoms. The need to differentiate between these two disease states is critical as the prognosis and management differ significantly. Physicians should be aware that C3GN recurrence is high in transplant patients and subsequently can lead to graft failure. We are hopeful that with the current understanding of the disease, better diagnostic and therapeutic agents will be developed in tackling the complement pathways disorders.
